# A modified primary culture method of rat pulmonary vein smooth muscle cells

**DOI:** 10.1186/s13019-023-02233-1

**Published:** 2023-04-17

**Authors:** Wenhui Huang, Hongjin Liu, Yichao Pan, Xueying Wang, Hongwei Yang, Danjie Wang, Jing Lin, Hui Zhang

**Affiliations:** 1 Critical Care Medicine, Union Hospital of Fujian Medical University, Fuzhou, Fujian Province 350001 P.R. China; 2 Anesthesiology Research Institute, the First Affiliated Hospital, Fujian Medical University, Fuzhou, Fujian Province 350004 P.R. China; 3 Department of Cardiovascular Surgery, Union Hospital of Fujian Medical University, Fuzhou, Fujian Province 350001 P.R. China; 4 Critical Care Medicine, Union Hospital of Fujian Medical University, NO.29 Xinquan Road, Gulou District, Fuzhou, Fujian, 350001 China

**Keywords:** Pulmonary vein smooth muscle cells, Pulmonary veins, Primary cell culture, Pulmonary hypertension due to left heart disease

## Abstract

**Background:**

Although the pressure of pulmonary vein increases before pulmonary artery in pulmonary hypertension due to left heart disease (PH-LHD), only a few studies have assessed pulmonary vein smooth muscle cells (PVSMCs) because of the lack of a simple and feasible isolation method.

**Methods:**

In this study, we introduced a simple method to obtain PVSMCs. Primary pulmonary veins were removed by puncture needle cannula guidance. Then, PVSMCs were cultured by the tissue explant method and purified by the differential adhesion method. The cells were characterized by hematoxylin-eosin (HE) staining, immunohistochemistry, western blotting, and immunofluorescence to observe the morphology and verify the expression of alpha-smooth muscle actin (α-SMA).

**Results:**

The HE staining results showed that the pulmonary vein media was thinner than the pulmonary artery, the intima and adventitia of the pulmonary vein were removed by this method, and the obtained cells with good activity exhibited morphological characteristics of smooth muscle cells. In addition, higher α-SMA expression was observed in the cells obtained by our isolation method than in the traditional method.

**Conclusion:**

This study established a simple and feasible method to isolate and culture PVSMCs that might facilitate the cytological experiments for PH-LHD.

## Introduction

Pulmonary hypertension (PH) due to left heart disease (PH-LHD) is the most common type of PH [1]. Increased pulmonary vein pressure occurs before the pulmonary artery and is caused by left heart disease, which ultimately leads to PH [2]. Currently, there are no effective treatment methods because of the unclear pathogenesis of PH-LHD. Pulmonary vascular remodeling is a critical mechanism of PH, and pulmonary artery smooth muscle cells (PASMCs) have been widely studied in pulmonary vascular remodeling research in vitro ^[3–4]^. The pulmonary vein has a thin medium and is easily contaminated when removed, making the pulmonary vein smooth muscle cells isolation method rather challenging; hence, PVSMCs have not been studied further.

The present study aimed to introduce a modified pulmonary vein isolation and primary PVSMC culture method to establish a foundation for studying pulmonary vein vascular remodeling in PH-LHD or other pulmonary diseases in the future.

## Methods

### Rats

Male Sprague-Dawley rats (body weight 300–400 g) were purchased from Shanghai SLAC Laboratory Animal Co. Ltd. (License No. SCXK (HU)2017 0005). All rats were housed in an environment with 12:12 h light-dark cycles at 22–24 ℃, and were given adequate food and water. All experimental protocols were consistent with the Institute of Laboratory Animal Resources, National Academy Press, Washington, DC1996.

### Drugs and materials

Pentobarbital sodium was purchased from Shanghai Rongbai Biological Technology Co. Ltd. DMEM (11,995,073), fetal bovine serum (FBS, 10,100,147), phosphate buffered saline (PBS) and penicillin-streptomycin (15,140,163) were obtained from Gibco (Carlsbad, CA, USA). Rabbit monoclonal anti-rat alpha-smooth muscle actin (α-SMA, 1:500, ab124964) and goat polyclonal anti-rabbit IgG-H&L (1:1000, ab150080) antibodies were purchased from Abcam. Other experimental materials were purchased from Thermo.

### Pulmonary vein and cell isolation

**Fig. 1:**
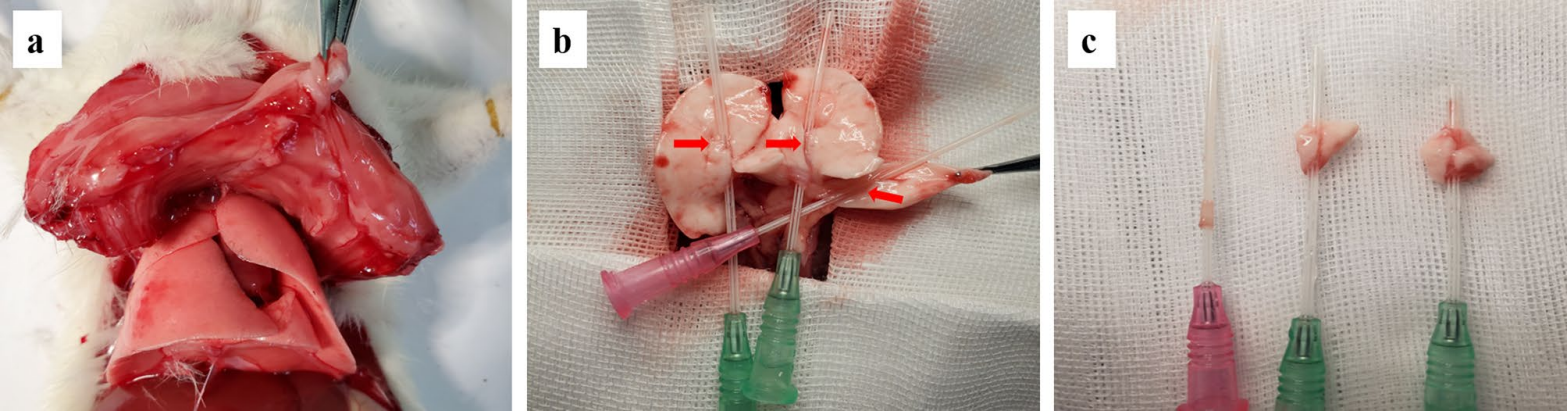
**a**: Rat thoracotomy. **b**: Puncture needle cannulas were inserted in the pulmonary veins (the red arrow). **c**: The connective tissue, pulmonary tissue, and adventitia of the pulmonary vein were removed (left one)

The rats were anesthetized with 2% pentobarbital sodium (50 mg/kg) by intraperitoneal injection and soaked in 75% alcohol for 10 min. Then, the animals were immobilized and disinfected, and the chest wall was excised in the middle to expose the heart and the lung. Because other pulmonary veins were too small for the entry of puncture needle cannulas, we chose the left superior pulmonary vein, right superior pulmonary vein and right inferior pulmonary vein in our study, located in the lung passing into the left atrium. The puncture needle cannulas (18 G) were inserted into the left upper and right lower pulmonary veins, and a puncture needle cannula (20 G) was inserted into the right upper pulmonary vein. The pulmonary vein was clipped along the puncture needle cannula (Fig. 1). The connective tissue and adventitia of the pulmonary vein were carefully removed under a microscope until the vascular ring became transparent. The pulmonary vein was rubbed 3–5 times on the cannula to remove the intima. Then, the cannula and the pulmonary vein were cut lengthwise and then the pulmonary vein was placed in sterile PBS. The pulmonary vein was cut into 1 mm pieces and transferred to a culture flask (25 cm^2^, 9–12 tissue pieces per bottle). The time from rat thoracotomy to placing the tissue in a culture flask was < 20 min. The culture flask was inverted, 3–5 mL of DMEM with high glucose and 20% FBS were added, and the cell culture incubated (37 ℃ under 5% CO_2_), The flask was turned over after 1 h. The cell growth around the tissue blocks was observed daily under an inverted microscope, and the culture medium was replaced every 3 days. At 80–90% confluency, the vascular tissue was removed, and the cells were serially passaged two times. To digest the cells, 1–2 ml of trypsin (0.25%, 37 ℃) was added to the culture flask and incubated for 3–5 min. When most of the cells were suspended or their morphology changed to rounded, 5 ml of DMEM containing 20% FBS was added to terminate the digestion. After 15 min of incubation, the cell suspension was centrifuged (5 min, 1500 RPM), and the supernatant was discarded. Finally, DMEM with 20% FBS was used to culture the cells.

PVSMCs were cultured to second generation, after trypsin digestion, 3–4 mL of culture solution was added immediately to terminate digestion, and the cells were gently blown with a straw repeatedly to make them free from the bottle wall and resuspended to form a single-cell suspension and incubated for 15 min, the impure cells based on the principle of fast adhesion rate of fibroblasts. Subsequently, the supernatant was collected by centrifugation (at 1500 rpm for 5 min), and the pellet was recultured.

The traditional method involves isolating distal pulmonary veins under a microscope and culturing PVSMCs by enzymatic digestion method, as described by Peng et al. [5] Also, the pulmonary artery was removed simultaneously under the microscope.

### Hematoxylin and eosin (HE) staining

The rats were anesthetized with 2% pentobarbital sodium (50 mg/kg) by intraperitoneal injection. The pulmonary veins and arteries were fixed in 4% paraformaldehyde for 24 h, dehydrated with ethanol, cleared with xylene, embedded in paraffin, and sliced into 4 μm thick sections. Then, the slices were dewaxed in xylene and stained with HE before observing under a microscope. The percentage of vascular medium smooth muscle thickness to vascular wall thickness was measured under the microscope.

### Immunohistochemical (IHC) analysis

The paraffin sections of the pulmonary vein were placed in an oven at 60 ℃ for 2 h, and the dehydrated in xylene for 15 min, 100% alcohol for 5 min, 85% alcohol for 5 min and 75% alcohol for 5 min. The antigen repair was carried out by heating the sections in citric acid buffer. Then, 50 µL of 3% hydrogen peroxide was added to the sections and incubated at 26 ℃ for 10 min. After blocking in goat serum for 30 min, the sections were incubated with rabbit monoclonal anti-rat α-SMA antibody (1:500) at 4 ℃ for 8 h, followed by goat anti-rabbit secondary antibody (1:1000) at 26 ℃ for 50 min. Finally, DAB was used for color development, and vascular smooth muscle cells were observed under a microscope (Nikon, Japan).

### Immunofluorescence analysis

Third-generation cells were seeded in 96-well plates at a density of 3000–5000 cells/ well and cultured for 1–2 days. Then, the cells were washed three times with PBS, fixed with 4% paraformaldehyde, permeabilized with 0.25% Triton-X 100, blocked with goat serum at 26 ℃ for 1 h, and incubated with α-SMA and CD31 primary antibodies at 4 ℃ for 8 h, and goat anti-rabbit secondary antibody at 26 ℃ for 1 h. Subsequently, the cells were treated with nuclear dye 594 (1 µg/mL) for 45 s, and observed under a confocal laser fluorescence microscope.

### Western blotting

The cells were lysed in RIPA buffer containing protease inhibitors and phosphorylase inhibitors on ice for 30 min. The supernatant was collected by centrifugation (12,000 rpm, 4 ℃ for 10 min). After the protein concentration was determined by the BCA method, the denatured proteins were separated by SDS-PAGE and transferred to a PVDF membrane for 45 min. Then, the membrane was blocked with 7% skim milk in TBS-T at 26 ℃ for 2 h and probed with rabbit monoclonal anti-rat α-SMA antibody at 4 ℃ for 8 h. Subsequently, the membrane was incubated with the secondary antibody for 2 h. Finally, the immunoreactive bands were developed by chemiluminescence instrument (ChemiDoc™ Touch System, Bio-Rad, USA). GAPDH was used as the internal control.

### Flow cytometry

PVSMCs were prepared as single-cell suspension at a concentration of 1 × 10^6^cells/mL and incubated with rabbit monoclonal anti-rat α-SMA antibody and staining buffer at 4 ℃ for 20 min. FACSCalibur flow cytometer was used for detection, and the results were analyzed by FACS Diva software.

### Statistical analysis

The experimental results are expressed as the mean ± standard error of the mean. A t-test was used for comparisons between two groups with homogeneity of variance, and one-way analysis of variance (ANOVA) was used for comparisons between multiple groups. *p* < 0.05 was considered as statistically difference. SPSS 25.0 was used for data analysis and GraphPad Prism 8.0 was used for data plotting.

## Results

### HE staining and IHC staining

**Fig. 2:**
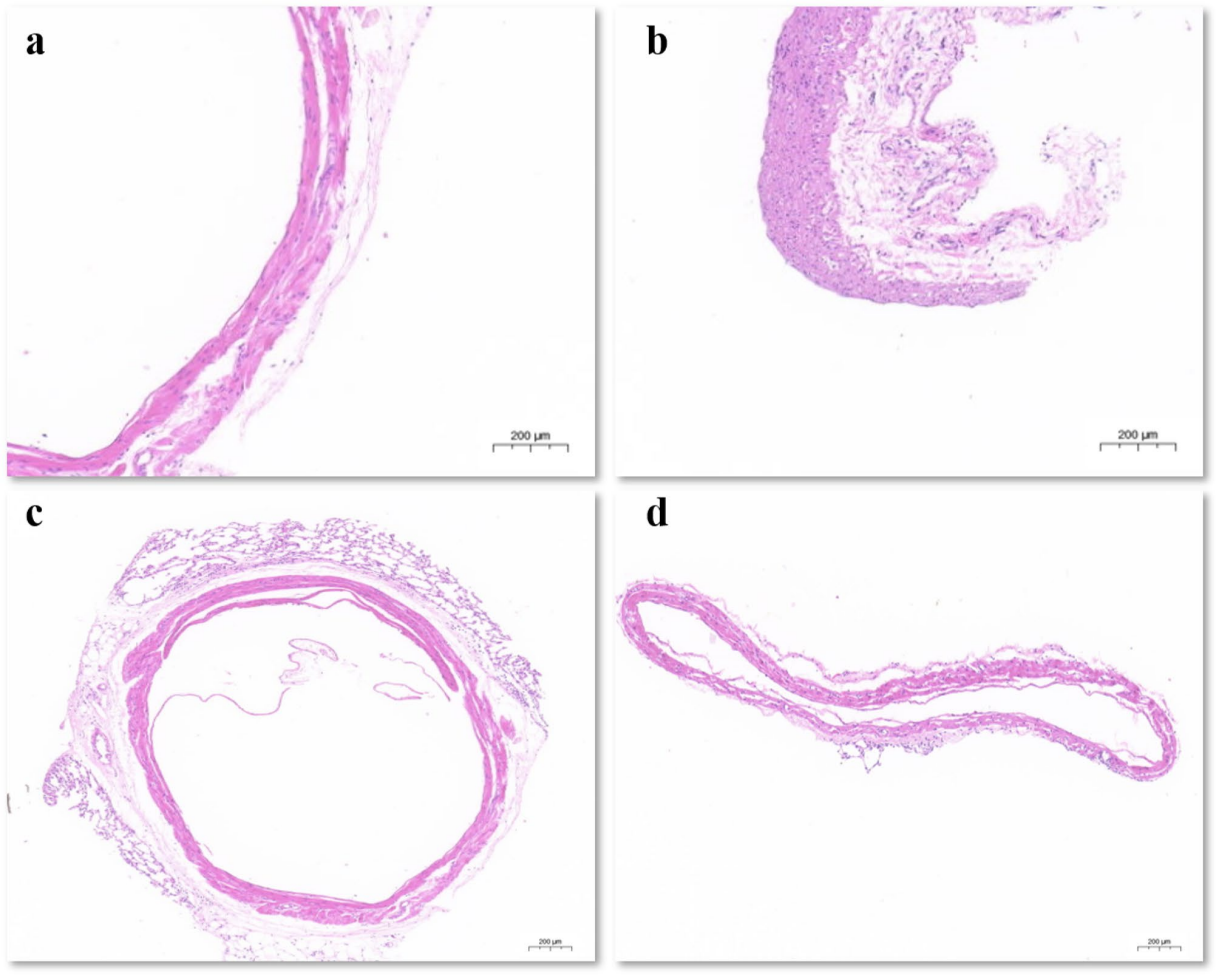
**a**: HE staining on the pulmonary vein. **b**: HE staining on pulmonary artery. **c**: HE staining on pulmonary vein before isolation. **d**: HE staining on pulmonary after isolation

HE staining was performed on the pulmonary veins and pulmonary arteries of rats, and the results showed that the proportion of pulmonary vein media to the vascular wall was smaller than that of pulmonary artery media (Fig. 2a and b). Also, the intima and adventitia of the pulmonary vein were removed by this method (Fig. 2c and d).

**Fig. 3:**
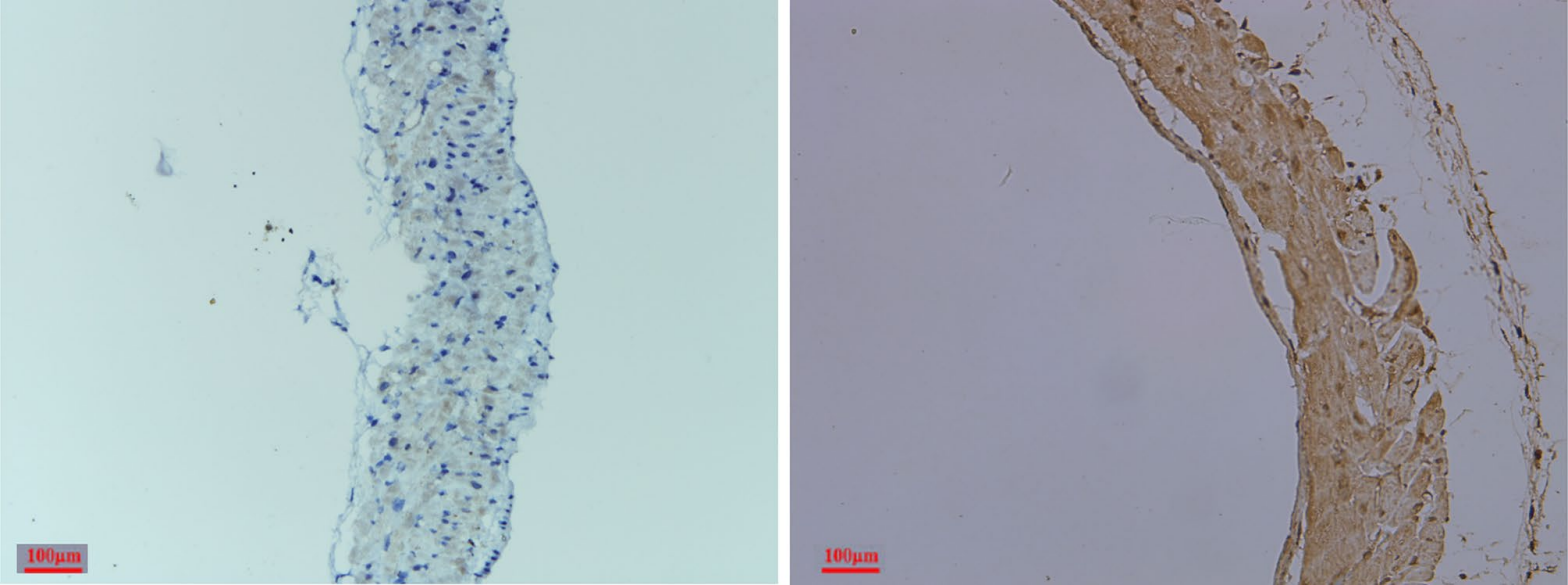
IHC experiments were performed to examine the levels of α-SMA in the pulmonary veins after isolation

Immunohistochemical staining showed that the remaining vascular cells expressed α-SMA (Fig. 3).

### Cell culture

**Fig. 4:**
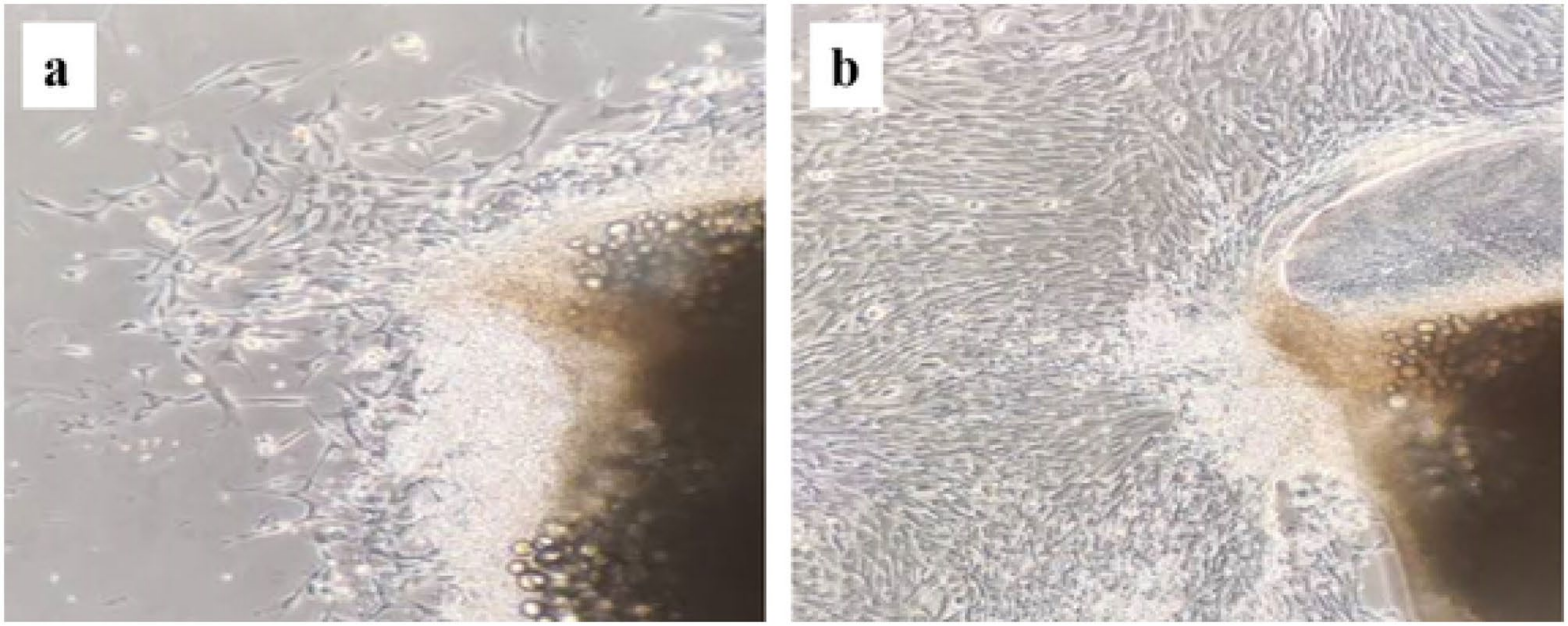
**a**: PVSMCs began to grow. **b**: PVSMCs overlapped as the number of cells increased

The tissue explant method was used to culture PVSMCs from second generation intrapulmonary vein branches, and the cells began to proliferate after 3 days, as observed under a microscope. The cells morphology was scattered and fusiform (Fig. 4a). PVSMCs overlapped as their number increased. At 70–80% confluency, some PVSMCs clustered, decreasing the PVSMC density of other parts, such that the characteristic “peak-valley” growth of smooth muscle cells appeared (Fig. 4b). The PVSMCs can fill the flask in 3–5 days. The cells were cultured to the third passage for subsequent experiments.

### Immunofluorescence

**Fig. 5:**
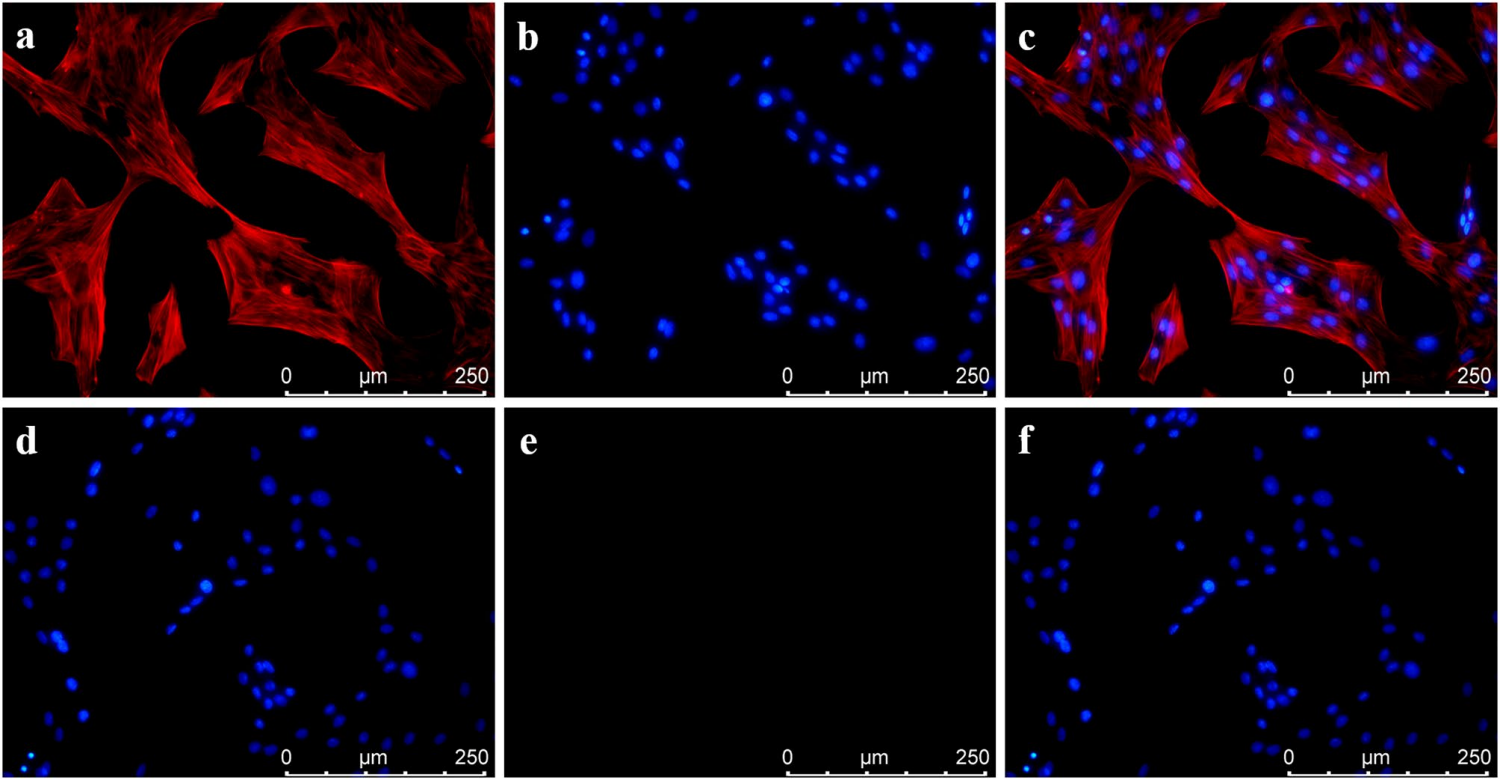
Identification of PVSMCs by morphology and immunofluorescence **a**, **b**, **c**: A large amount of α-SMA was observed under a fluorescence microscope. The purity of PVSMCs was determined to be high based on the association between α-SMA (stained red) and the cell nucleus (stained blue). **d**, **e**, **f**: The immunofluorescence results showed that α-SMA was negative in the control PVSMCs

Immunofluorescence analysis was performed on PVSMCs removed by our method. The fibers were parallel to each other in PVSMCs (Fig. 5). Overlapping growth occurred when the cell density was high, and the fibers around the nucleus in PVSMCs stained strongly for α-SMA (shown in red, Fig. 5a). Nuclear staining with DAPI showed blue fluorescence (Fig. 5b). The merged the images of the fibers and the nuclei revealed the fibers corresponding to the nuclei (Fig. 5c). The control group was not treated with the α-SMA antibody, and immunofluorescence results indicated that α-SMA was negative control in PVSMCs (Fig. 5d, e and f).

**Fig. 6:**
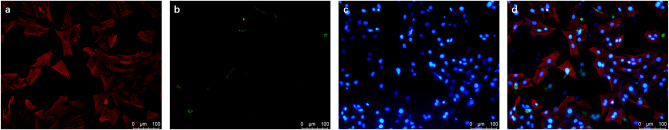
Double-immunofluorescence labeling method was executed on PVSMCs to examine α-SMA (shown in red, Fig. 6a) and CD31 (shown in green, Fig. 6a)

Double-immunofluorescence labeling method showed that the fibers surrounding the nucleus in PVSMCs were stained strongly for α-SMA (shown in red, Fig. 6a) with CD31 low expression (shown in green, Fig. 6b). The nucleus was stained with DAPI (Fig. 6c), and merged images of the fibers and the nuclei are illustrated in Fig. 6d.

### Western blotting

**Fig. 7:**
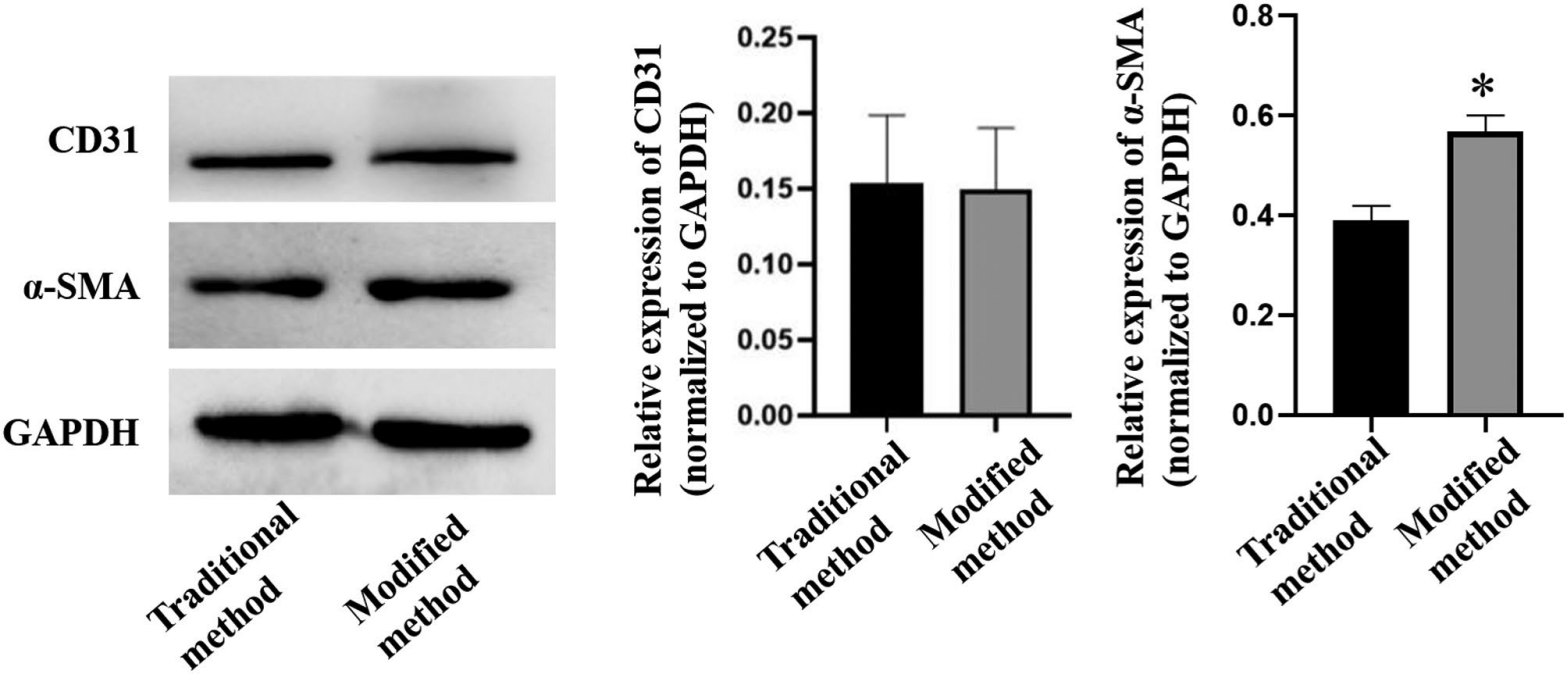
Western blotting was performed on the pulmonary vein in traditional and modified methods to examine α-SMA and CD31. *p < 0.05 vs. the traditional method

The Western blot results showed that α-SMA expression in PVSMCs removed by our method was higher than in the traditional method (*p <* 0.05). However, no significant difference was observed in CD31 expression in PVSMCs removed by our method and traditional methods (*p* > 0.05, Fig. 7), which indicating that the PVSMCs obtained by our method had high purity, and the degree of endothelial cell removal was similar to that obtained by traditional methods.

### Flow cytometry

**Fig. 8:**
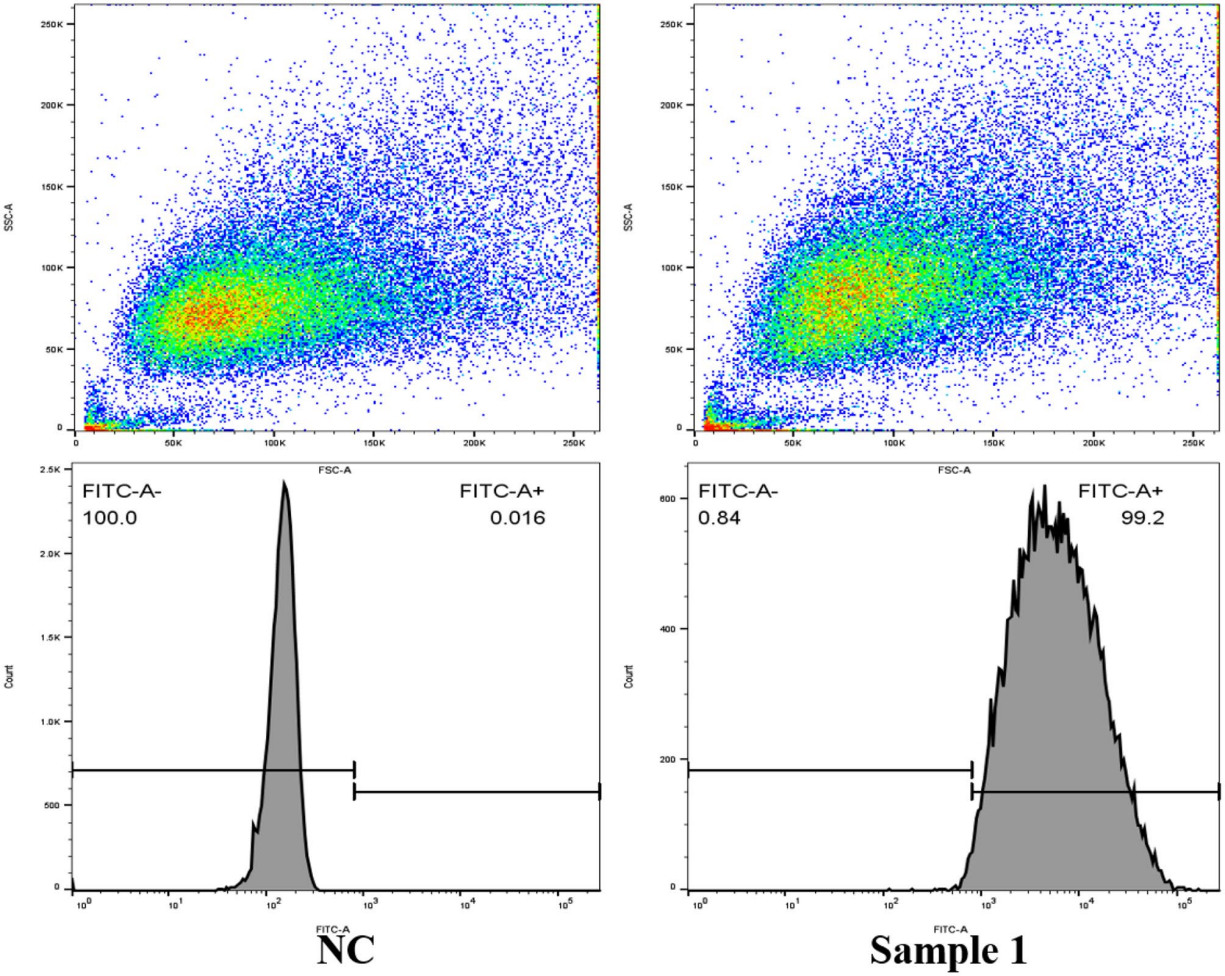
Flow cytometry on PVSMCs to examine α-SMA

The flow cytometry results showed that > 99% cells expressed α-SMA (*p* < 0.05, Fig. 8).

## Discussion

PH-LHD is the most common type of PH in patients. Pulmonary vein pressure is the first to increase, eventually causing PH [6]. Most studies have focused on pulmonary arterial hypertension. However, the current study showed early expression of inflammatory factors and TGF-β1 in the pulmonary vein than in the pulmonary artery of rats. Moreover, the expression levels were increased after a mechanical stretch in the pulmonary vein [7]. Frid et al. found that inflammatory reactions were related to PH [8]. In addition, Ping et al. demonstrated early vascular changes (thickened media and narrow lumen) in the pulmonary veins than in the pulmonary artery in the PH-LHD rat model [9].

In this study, we developed a method to isolate PVSMCs. According to the anatomy, the pulmonary vein is connected to the left atrium and separated from the second pulmonary vein branches with the aid of a puncture needle cannula. Presently, most methods obtain pulmonary veins or arteries by removing the distal pulmonary vascular [10], but not the main pulmonary vascular, especially via the enzymatic digestion method [11]; hence, the obtained vein is thin with a low weight. Compared to the vascular media of pulmonary arteries, that of the pulmonary vein was thinner, making it difficult to distinguish the intima from the adventitia [5]. Therefore, our method used the second pulmonary vein branch to obtain more tissue and made the tissue explant method easy. In addition, HE staining showed that due to the use of the puncture cannula, the intima and adventitia of the pulmonary veins were removed more thoroughly than the traditional method. The remaining vascular media clearly expressed α-SMA with low expression of cd31, as shown by IHC. Therefore, the remaining vascular medium was VSMCs. Moreover, the rate of endothelial cells by the two methods was similar. Our method to isolate the pulmonary vein greatly reduced the exposure time of the pulmonary vein in bacterial conditions (< 20 min), such that the probability of cell contamination was reduced.

PVSMCs were obtained by the pulmonary vein tissue explant method, and the fibroblast was removed by the velocity differential adhesion method. The obtained cells showed obvious morphological characteristics of VSMC, and cell fusion appeared after the cells grew to a certain density. Finally, the characteristic “peak-valley” growth of VSMCs appeared. Almost all PVSMCs removed by our method expressed α-SMA by immunofluorescence.

Moreover, the cell fibers were similar to those of VSMCs in previous studies, and the PVSMCs proliferated rapidly to about 100% in 3–5 days. In addition, PVSMCs removed by our method had higher α-SMA expression than the traditional methods. These results confirmed that the cells acquired and cultured by our method were PVSMCs in good condition.

## Conclusions

In this study, puncture needle cannulas were used to guide the pulmonary vein tissue isolation under a microscope, and the differential adhesion method was used to purify PVSMCs with a strong proliferation ability and high purity. Although the preliminary outcomes are encouraging, further trials are necessary to evaluate the efficacy of this simple primary culture method.

## Data Availability

Data used for this study are available upon request.
