# Correction to: A modified primary culture method of rat pulmonary vein smooth muscle cells

**DOI:** 10.1186/s13019-023-02306-1

**Published:** 2023-06-30

**Authors:** Wenhui Huang, Hongjin Liu, Yichao Pan, Xueying Wang, Hongwei Yang, Danjie Wang, Jing Lin, Hui Zhang

**Affiliations:** 1 Critical Care Medicine, Union Hospital of Fujian Medical University, Fuzhou, Fujian Province 350001 P.R. China; 2 Anesthesiology Research Institute, the First Affiliated Hospital, Fujian Medical University, Fuzhou, Fujian Province 350004 P.R. China; 3 Department of Cardiovascular Surgery, Union Hospital of Fujian Medical University, Fuzhou, Fujian Province 350001 P.R. China; 4 Critical Care Medicine, Union Hospital of Fujian Medical University, NO.29 Xinquan Road, Gulou District, Fuzhou, Fujian 350001 China

Following publication of the original article [1], in this article all figures had been interchanged and now it has been corrected.

The original article has been corrected.
